# Lipids in Clinical Nutrition and Health: Narrative Review and Dietary Recommendations

**DOI:** 10.3390/foods14030473

**Published:** 2025-02-01

**Authors:** Adrian Frydrych, Kamil Kulita, Kamil Jurowski, Wojciech Piekoszewski

**Affiliations:** 1 Laboratory of Innovative Toxicological Research and Analyses, Faculty of Medicine, Medical College, University of Rzeszów, Al. mjr. W. Kopisto 2a, 35-959 Rzeszów, Poland; afrydrych@ur.edu.pl (A.F.); toksykologia@ur.edu.pl (K.J.); 2 Toxicological Science Club ‘Paracelsus’, Faculty of Medicine, Medical College, University of Rzeszów, Al. mjr. W. Kopisto 2a, 35-959 Rzeszów, Poland; kk119465@stud.ur.edu.pl; 3 Department of Regulatory and Forensic Toxicology, Institute of Medical Expertise, Łódź, ul. Aleksandrowska 67/93, 91-205 Łódź, Poland; 4 Laboratory of High Resolution of Mass Spectrometry, Faculty of Chemistry, Jagiellonian University, R. Ingardena 3, 30-060 Krakow, Poland

**Keywords:** lipids, clinical nutrition, dietary fats, omega-3 fatty acids, chronic disease management, lipid metabolism

## Abstract

Lipids are essential components of human health, serving as critical structural elements of cell membranes, energy sources, and precursors for bioactive molecules. This narrative review aims to examine the multifaceted roles of lipids in clinical nutrition and health, focusing on their impact on chronic disease prevention, management, and the potential of lipid-based therapies. A narrative review was conducted utilizing Scopus, Google Scholar, and Web of Science databases. Key terms such as lipids, dietary fats, and cholesterol were used to identify and analyze relevant studies. A total of 145 articles meeting inclusion criteria were reviewed for their insights into lipid metabolism, dietary sources, and clinical implications. The analysis highlighted the metabolic significance of various lipid classes—saturated, monounsaturated, and polyunsaturated fatty acids—along with evidence-based recommendations for their dietary intake. Lipids were shown to play a pivotal role in managing chronic diseases such as cardiovascular disease, obesity, and metabolic syndrome. Emerging therapies, including omega-3 fatty acids and medium-chain triglycerides, demonstrated potential benefits in clinical practice. By synthesizing current knowledge, this narrative review provides healthcare professionals with an updated understanding of the roles of lipids in clinical nutrition. The findings emphasize the importance of tailored dietary interventions and lipid-based therapies in optimizing health and managing chronic diseases effectively. Additionally, this review successfully presents practical dietary recommendations to guide clinical practice.

## 1. Introduction

Lipids are vital components of human nutrition, providing a primary energy source and playing key roles in numerous biological functions. They are crucial for the structure and function of cell membranes, serve as signaling molecules, and participate in the regulation of gene expression and protein synthesis [1,2]. Dietary lipids, such as fatty acids, carotenoids, and phytosterols, have been demonstrated to prevent or treat chronic diseases owing to their bioactive properties [1]. The type of dietary lipids consumed can have a significant impact on health outcomes. Saturated fats have been linked to elevated levels of lipotoxic lipids and the aggregation of low-density lipoprotein (LDL), both of which are risk factors for cardiovascular diseases [3]. In contrast, polyunsaturated fatty acids (PUFAs), including omega-3 fatty acids, have been associated with anti-inflammatory effects and a reduced risk of cardiovascular risk [2]. These fatty acids are essential for proper neural and retinal development, especially in infants and young children [2]. Lipidomics, the extensive study of lipids in biological systems, has become an important tool in nutrition research. It enables the detailed analysis of lipid profiles and their interactions with diet and metabolism, offering valuable insights for developing personalized nutrition strategies [3,4]. This approach can aid in customizing dietary recommendations based on individual lipid responses, potentially enhancing health outcomes [4]. In clinical settings, lipids are utilized in parenteral nutrition (PN) to supply essential fatty acids and decrease the dependence on dextrose for non-protein calories. Alternative lipid formulations, such as those combining olive, soy, and fish oils, have demonstrated reduced inflammatory properties and improved clinical outcomes compared to traditional soybean-oil-based emulsions [5]. Additionally, lipids contribute to nutritional support for malnourished cancer patients, offering potential benefits in enhancing nutritional status and improving treatment efficacy [6].

When discussing lipids, it is worth highlighting their role in preventing hospital malnutrition, as emphasized in numerous scientific studies [7]. Fats are key components of various specialized nutritional preparations used during hospitalization, as discussed in our previous studies [8,9,10,11]. In hospital settings, particularly for patients with conditions such as cancer, lipids play an essential role in nutritional support. They aid in managing malnutrition by providing essential energy and nutrients, although their application may be limited by sensory and physicochemical properties [12]. In patients with acute myocardial infarction, the relationship between lipid levels and outcomes is complex, as malnutrition may influence the effectiveness of lipid management strategies [13].

Lipids are essential to human health and nutrition, impacting a variety of physiological processes and disease outcomes. Advances in lipidomics and nutrigenomics are enabling more personalized and effective dietary interventions. A deeper understanding of the complex roles of different lipids can lead to improved health strategies and better management of chronic diseases. In this review, we provide a comprehensive overview of the role and importance of lipids in human nutrition, both in health and disease. We discuss, among other topics, the scientific literature and the guidelines of the European Society for Clinical Nutrition and Metabolism (ESPEN).

### Aims

The primary objective of this study was to provide a comprehensive overview of the role and importance of lipids in human nutrition, with a particular focus on their physiological functions, contributions to health, and implications in the prevention and management of chronic diseases. The secondary objective was to explore recent advancements in lipidomics and nutrigenomics, highlighting their potential in developing personalized dietary interventions and improving health outcomes. Additionally, this study aimed to integrate insights from the scientific literature and the guidelines of the ESPEN to present evidence-based recommendations for clinical and nutritional practice.

## 2. Materials and Methods

A multifaceted data collection methodology was employed to thoroughly examine various aspects of lipids in clinical nutrition and health. The three primary repositories for locating published references on this topic—Scopus, Google Scholar, and Web of Science—were utilized to critically analyze key lipids in the context of nutrition and health.

Different combinations of the following key terms were used: lipids, cholesterol, dietary lipids, fats, and combinations of these terms. The study selection process involved two steps: (1) screening by title and abstract and (2) full-text review. Title and abstract screening were conducted independently by each author, at separate time points. Studies were clearly identified as included or excluded after defining the problem statement. Those meeting the eligibility criteria were retained for further screening, while studies irrelevant to the problem statement or matching exclusion criteria were removed. We analyzed all available sources (*n* = 145 articles and related content). Only articles relevant to the role of lipids in clinical nutrition and health were selected. Subsequently, the authors performed an in-depth full-text review of all relevant articles. We employed a strategic blend of advanced digital tools, including Covidence, Consensus, and SysRev, to facilitate the identification, selection, and review of the key literature on lipids. The integration of these platforms was pivotal in ensuring an organized and efficient review process, enabling us to effectively manage the complexity of synthesizing extensive data from diverse sources. We evaluated the studies referenced in our work using the SANRA scale [14]. The mean score for all the studies included in our review was 8.25. For detailed scoring, refer to the Supplementary Materials (Table S1).

## 3. Results and Discussion

### 3.1. Definition and Classification of Lipids

Triglycerides are essential in nutrition and health, influencing energy storage, metabolic processes, and disease susceptibility. As the body’s main form of energy storage, they support numerous physiological functions. Nonetheless, high triglyceride levels are linked to various health concerns, such as cardiovascular diseases and metabolic conditions. Triglycerides serve as a key energy source, contributing approximately 30–40% of the total energy intake in a standard Western diet. They are highly efficient in digestion and absorption, with the body utilizing more than 95% of the triglycerides consumed [15]. They include essential polyunsaturated fatty acids that act as precursors for critical lipid-derived mediators, such as prostaglandins and leukotrienes, which play a key role in regulating various cellular processes [15]. High triglyceride levels are associated with a greater risk of cardiovascular diseases (CVDs). They promote the development of atherogenic remnant-like particles and are linked to conditions such as non-alcoholic fatty liver disease and pancreatitis [16,17]. Elevated triglyceride levels are a hallmark of metabolic syndrome, characterized by glucose intolerance and dyslipidemia. This syndrome significantly raises the likelihood of developing atherosclerotic cardiovascular diseases [18,19]. The prolonged consumption of high-fat diets, which boost triglyceride intake, can result in obesity and associated metabolic disorders. Controlling the absorption of dietary triglycerides presents a promising therapeutic approach for managing these conditions [19,20]. Modifying macronutrient distribution, such as lowering fat intake or adopting low-carbohydrate diets, can aid in controlling triglyceride (TG) levels. The Mediterranean diet and omega-3 supplements have proven effective in reducing triglyceride levels [16]. MCTs are metabolized more quickly than long-chain triglycerides (LCTs) and may aid in preventing obesity. However, their impact on fat accumulation and metabolic health is complex and requires further research [20,21]. Blocking triglyceride absorption through dietary components or medications can lower blood triglyceride levels and reduce energy intake, potentially helping treat obesity and hypercholesterolemia [22]. Triglycerides play a crucial role in energy storage and metabolic processes, but elevated levels present substantial health risks. Proper management through dietary changes and medical treatments can help reduce these risks and enhance overall health. Diacylglycerol (DAG) is a type of functional oil known for its physiological properties, including the ability to reduce fat accumulation, prevent systemic inflammatory conditions, and support cardiovascular health, along with numerous other health advantages. DAG acts as a key intermediate in lipid metabolism, playing a role in the production of triacylglycerol (TAG) and PA through the enzymatic actions of diacylglycerol acyltransferases (DGATs) and diacylglycerol kinases (DGKs). This mechanism is crucial for preserving lipid homeostasis and regulating cellular energy balance [23]. In the food industry, DAG is utilized as a functional oil with potential health advantages, including reducing body fat accumulation and lowering cholesterol levels. However, its safety as a cooking oil remains a topic of debate due to concerns about potential carcinogenic risks [24].

Phospholipids are vital for nutrition and health, providing numerous benefits, including lowering cholesterol absorption, enhancing liver function, and reducing the risk of cardiovascular diseases. They are key components of cell membranes and participate in a wide range of biological processes. Phospholipids aid in reducing cholesterol absorption and enhancing liver function, which in turn helps lower the risk of cardiovascular diseases [25]. They provide choline and essential fatty acids, both of which are crucial for numerous cellular functions and overall health [26]. Phospholipids have been shown to enhance cognitive functions, such as learning and memory, and may help alleviate the impact of stress and aging on brain health [27]. Phospholipids are key structural components of cell and organelle membranes, affecting membrane-bound proteins and overall cell function [28]. They act as precursors to prostaglandins and other signaling molecules, regulating gene expression and cellular processes [28]. In young organisms, dietary phospholipids improve the efficiency of lipid transport from the gut to other parts of the body, potentially by enhancing lipoprotein synthesis [29]. Phospholipids are present in a variety of foods, such as milk, vegetable oils, egg yolk, and meat [25,30]. Marine sources also offer phospholipids with anti-inflammatory properties. In the small intestine, phospholipids are absorbed and play a crucial role in the uptake of dietary lipids [26]. Dietary phospholipids, particularly those from dairy and marine sources, have anti-inflammatory properties that can help manage inflammation-related disorders [30]. Supplementing infant diets with milk phospholipids has demonstrated notable benefits for gut health and brain development [28,31]. Phospholipids are crucial for supporting various aspects of human health, including cardiovascular and liver functions, cognitive health, and inflammation management. As key components of cell membranes, they play a significant role in nutrient absorption and cellular signaling. Including them in a balanced diet can enhance overall well-being and help prevent disease.

Sterols, especially plant sterols, and stanols are important in human nutrition and health, mainly because of their ability to reduce cholesterol levels and potentially provide additional health benefits. Plant sterols and stanols lower serum LDL cholesterol by competing with dietary and biliary cholesterol for absorption in the intestines. This competition helps reduce the amount of cholesterol absorbed into the bloodstream [30,32,33,34,35]. Consuming 2–3 g of plant sterols or stanols daily can reduce LDL cholesterol levels by 9–12% [34]. Both sterols and stanols are equally effective in their free, unesterified form in lowering plasma LDL cholesterol [35]. By reducing LDL cholesterol levels, plant sterols and stanols help decrease the risk of cardiovascular diseases [34,36]. Emerging evidence indicates that plant sterols and stanols may offer additional health benefits, including potential roles in immunology, hepatology, pulmonology, and gastroenterology. They may also have an impact on conditions such as non-alcoholic steatohepatitis (NASH), inflammatory bowel diseases (IBDs), and allergic asthma [36]. Plant sterols and stanols inhibit cholesterol absorption in the intestines by competing for incorporation into mixed micelles and chylomicrons [37,38]. They may influence proteins involved in cholesterol metabolism, such as ATP-binding cassette transporters (ABCA1 and ABCG5/G8), although their cholesterol-lowering effect occurs independently of these transporters [37]. Plant sterols and stanols may also influence cholesterol esterification, lipoprotein assembly, and the clearance of apolipoprotein B100-containing lipoproteins [33]. Common dietary sources of plant sterols include vegetable oils, cereals, nuts, and vegetables. These foods are high in sterol esters, which are the primary form of plant sterols ingested [32]. Foods fortified with plant sterols and stanols are commonly used as a therapeutic dietary approach to manage hypercholesterolemia [34]. Plant sterols and stanols are effective in reducing LDL cholesterol, thereby lowering the risk of cardiovascular diseases. They work by interfering with cholesterol absorption in the intestines and may also regulate cholesterol metabolism at the molecular level. Furthermore, emerging research indicates that they may offer additional health benefits beyond cholesterol management.

**Figure 1:**
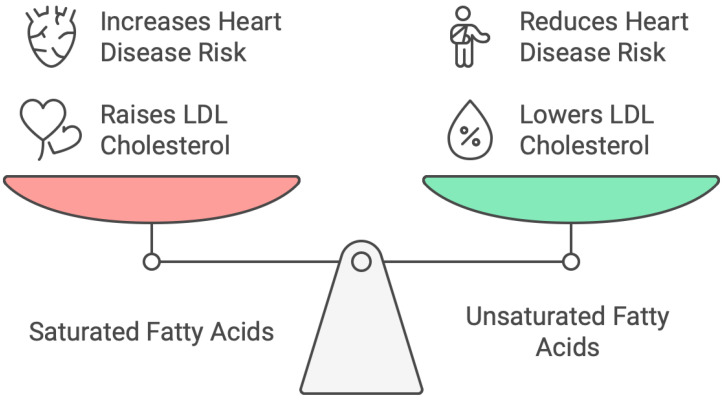
Benefits and risks of consuming fats.

As essential parts of lipids, fatty acids are vital for human nutrition and general well-being. Saturated, unsaturated, and trans fatty acids are the three primary categories into which these fatty acids fall; each has unique physiological effects and health concerns. Generally speaking, animal items like meat, butter, and cheese, as well as certain plant-based oils like coconut and palm oil, include saturated fatty acids (SFAs). LDL cholesterol, a major risk factor for CVD, has long been linked to elevated blood levels of SFAs. The high consumption of SFAs has been associated with a higher risk of coronary heart disease (CHD), according to a study by Siri-Tarino et al. [39]. However, more recent research has questioned this association [40]. Nevertheless, to lower the risk of CVD, health organizations like the American Heart Association advise limiting SFA intake [41]. Monounsaturated fatty acids (MUFAs) and PUFAs are the two main categories of unsaturated fatty acids. Nuts, seeds, fatty fish, and plant-based oils (canola and olive) are the main sources of these fats. Because they can lower inflammation and LDL cholesterol, unsaturated fats are thought to be good for heart health. According to Sacks et al.’s [42] systematic study, substituting unsaturated fats for saturated fats reduces the incidence of cardiovascular events and improves general health. According to Kris-Etherton et al. [43], omega-3 fatty acids, a form of PUFA that can be found in walnuts, flaxseeds, and fatty fish, are especially well known for their anti-inflammatory properties and ability to lower the risk of cardiovascular disease. Trans fatty acids are frequently found in processed and fried meals and are mostly prevalent in partially hydrogenated oils. Trans fats have been repeatedly associated with negative health outcomes, especially when it comes to raising LDL cholesterol and lowering HDL (high-density lipoprotein) cholesterol, both of which increase the risk of cardiovascular illnesses. TFA (trans monounsaturated fatty acid) consumption is strongly linked to an elevated risk of coronary heart disease, according to a meta-analysis conducted by Mozaffarian et al. [44]. Many nations have put laws in place to limit or completely ban trans fats in food items because of these negative consequences. Fatty acids are vital nutrients that have a big impact on people’s health. The excessive consumption of saturated and trans fats can raise the risk of cardiovascular disorders, but unsaturated fats are typically good for the heart and reduce inflammation. Lipid profiles and general health can benefit from substituting unhealthy fats with healthier unsaturated fats, especially omega-3 fatty acids. Therefore, fatty acid consumption must be carefully managed and regulated to prevent chronic diseases and support long-term health. Figure 1 presents the health benefits and risks associated with fat consumption.

### 3.2. Biological Role of Lipids

Lipids are fundamental to human biology, functioning as key elements in numerous physiological processes. They contribute to energy storage, facilitate cellular communication, and maintain the structural stability of cell membranes. Lipids are essential components of cellular membranes, providing the structural foundation that defines cell boundaries and organelle compartments. Phospholipids and sphingolipids play a crucial role in preserving membrane integrity and fluidity [45,46,47]. Lipids play a key role in membrane dynamics, impacting processes like cell division and morphological alterations [47].

Lipids serve as essential building blocks of biological membranes, offering support for membrane proteins and playing a pivotal role in determining membrane properties and organization [48,49,50]. Lipids, especially in the form of triacylglycerols, act as primary energy storage molecules, offering a concentrated energy source that can be mobilized when required [46,51]. Lipids function as bioactive signaling molecules, facilitating both intra- and inter-cellular communication. They play a role in numerous signaling pathways that govern cellular processes [49,52,53]. Lipids are crucial in modulating immune responses, affecting the activation and differentiation of immune cells. Their impact can be either beneficial or harmful, depending on the context, such as during acute infections or chronic inflammatory conditions [54]. Imbalances in lipid metabolism are associated with a variety of diseases, including cardiovascular, metabolic, neurodegenerative, and cancer-related conditions. Lipids also play a role in the development of diseases such as atherosclerosis and Niemann–Pick disease [55,56]. Lipid metabolism is involved in the regulation of aging and lifespan, with some lipid-based interventions demonstrating the potential to extend lifespan in model organisms [57]. Lipids are essential for human health, serving as structural elements, energy stores, and signaling molecules. They are key to immune regulation and are linked to various diseases when their metabolism is disrupted.

### 3.3. Dietary Sources of Lipids

The primary dietary sources of lipids are vegetable oils, fish oils, nuts, meats, sweets, and cookies. The latter (meats, especially pork, and beef, as well as sweets and cookies) will predominantly serve as a source of unfavorable saturated fatty acids. These sources supply essential fatty acids and energy, serving a vital role in human nutrition. Vegetable oils, including soybean, palm, linseed, rapeseed, and coconut oils, are major dietary lipid sources. Extracted from plants, these oils are typically refined to eliminate impurities and improve their quality. The refining process, tailored to the type of oil and extraction method, enhances shelf life and consumer appeal. Widely utilized in cooking and food manufacturing, vegetable oils are a key source of mono- and polyunsaturated fats [58]. Fish oils are an important dietary lipid source, abundant in omega-3 fatty acids such as EPA and DHA. Extracted from fish, they are valued for their health benefits, including anti-inflammatory effects and support for cardiovascular health. To ensure purity and remove contaminants, fish oils typically undergo a refining process. They are consumed as dietary supplements or added to a variety of food products [59]. Nuts, including almonds, walnuts, and peanuts, are rich natural lipid sources. They offer a combination of mono- and polyunsaturated fats, along with valuable nutrients such as protein, fiber, and vitamins. Nuts can be eaten whole, processed into nut butter, or used as ingredients in various dishes, serving as a healthy lipid source with minimal processing required [58]. Animal studies, including those with weaned piglets, have demonstrated that different lipid sources can influence growth performance and nutrient digestibility. For example, blends of fish, palm, and rice oils have been shown to enhance digestibility and promote better intestinal health compared to soybean oil [60]. In fish, replacing a substantial portion of fish oil with vegetable oils does not negatively affect growth performance or lipid metabolism. This substitution can reduce cholesterol levels without significantly influencing lipogenesis or lipid transport [61]. Sweet baked goods and chocolate products frequently contain significant amounts of saturated fatty acids, predominantly palmitic and oleic acids [62]. Research has shown that in some cases, saturated fats make up over 44% of the total fatty acids in these items. Similarly, Iranian traditional sweets have been found to have an average saturated fatty acid content of 38.6% of total fat, with certain sweets containing as much as 92.4% [63]. Meat fat is mainly composed of monounsaturated and saturated fatty acids, with oleic, palmitic, and stearic acids being the predominant types [64,65]. Ruminant animals, such as cattle and sheep, have higher levels of saturated fats due to the process of biohydrogenation in the rumen. In contrast, the fatty acid composition of monogastric animals, like pigs, more closely mirrors their diet [64,66]. A high intake of saturated fats from meat is linked to obesity, type 2 diabetes, and cardiovascular diseases. Therefore, the moderation of and preference for lean meats are recommended [65].

### 3.4. Lipid Metabolism

Lipid metabolism is a complex process that we can divide into several stages, which include complex mechanisms. First of all, lipid metabolism includes the processes of synthesis and degradation. Disorders in the background of one of these processes can lead to a disturbance of the homeostasis of the body and, thus, to many complications [67]. The functions of lipids are diverse depending on the organ in question, and the impact of this group of compounds will affect different processes. Lipids are the basic structure of membranes, cell membranes, a structure that serves as a barrier between the interior of the cell and its surroundings. The main component building cell membranes are phospholipids. Phospholipids are esters of glycerol and phosphoric acid and are fatty acids (FAs). In addition, in the composition of phospholipids, we find choline and ethanolamine, containing nitrogen. Remarkably, most phospholipids contain two-chain FAs. Usually, one consists of a saturated acid chain and the other of an unsaturated acid. Importantly, given the basic building block of cell membranes, the composition of phospholipids remains not insignificant, because the most unsaturated fatty acids containing more than 60% are contained in phosphatidylethanolamine, while more than 30% of long-chain fatty acids are contained in sphingomyelin. Phospholipids contain more unsaturated fatty acids compared to triacylglycerols [68]. Knowledge and understanding of the mechanisms of lipid interactions seem to be crucial for many metabolic disorders, as well as cancer. Lipids play a key role in cell signaling and are involved in hormone formation. In addition to metabolic and hormonal effects, lipids are an important source of energy as a result of beta-oxidation, dependent on mitochondria (the body’s energy factory). In addition, they participate in the catabolism of the Krebs cycle, one of the stages of aerobic cellular respiration [69]. As we said earlier, lipid metabolism includes catabolic processes—the creation of energy—and anabolic processes—the formation of various lipid species. Speaking of lipid metabolism, it is worth noting triglycerides. This is the main form in which we store fats; moreover, elevated TG levels are associated with many metabolic diseases: atherosclerosis, ischemic heart disease, metabolic syndrome, and hypertension. Lipids are transported into proteins, involving specialized transport proteins, fatty acid transport proteins (FATPs), and cluster of differentiation (CD36) [69]. When cells require lipids, FAs are transported to the mitochondria, with the participation of membrane proteins. This is followed by the oxidation of lipids in the mitochondria to produce acetyl-CoA, which in turn is necessary for the production of adenosine triphosphate (ATP). Importantly, acetyl-CoA can contribute to increased levels of gene expression via histone acetylation. Mitochondria are not the only place where FA oxidation occurs; the mentioned process also takes place in peroxisomes, thus affecting tricarboxylic acid (TCA) metabolism [70]. Citrate, on the other hand, is synthesized during the Krebs cycle and then transferred to the cytoplasm, primarily for de novo lipogenesis (synthesis of cholesterol and fatty acids). In addition, fatty acids can be synthesized from malonyl-CoA, which represents the key step in fatty acid synthesis. The previously mentioned acetyl-CoA and malonyl-CoA are used to produce palmitate, which is subsequently elongated to form PUFA and MUFA. Palmitate is then converted into diglyceride (DAG) and TG. It is important to note that different metabolic pathways are interconnected to regulate the metabolic state of the cell. Furthermore, the role of mitochondria in regulating lipid metabolism, both in anabolic and catabolic processes, should be emphasized. The production of energy molecules in the form of ATP from fats is twice as high as that from carbohydrates [71]. Moreover, in order for the body to obtain energy from lipids through oxidation in the mitochondria, it is necessary to break down fatty acids in the process of beta-oxidation, which takes place in the mitochondrial matrix. During beta-oxidation, the tails of fatty acids are broken down into a series of two-carbon units, which then combine with coenzyme A, thereby forming acetyl-CoA. Acetyl-CoA is an essential fuel source for the TCA cycle. Adequate amounts provide the foundation for adaptation in skeletal muscle cells, which are rich in mitochondria and enzymes necessary for proper metabolism. Furthermore, more than a dozen studies have shown that the body’s capacity to oxidize fatty acids relative to its metabolic needs, as well as their proper utilization, is a key element for maintaining the homeostasis of lipid and glucose metabolism, as well as insulin sensitivity [72].

### 3.5. Lipids and Energy Homeostasis

Lipids, as energy storage molecules, are key to maintaining proper energy metabolism in the body, and thus their correct concentration is essential for maintaining the body’s homeostasis. Disturbances in the processes of synthesis, or degradation, will contribute to the disruption of specific functions of the body and prevent the maintenance of energy homeostasis. The manifestation of disorders of energy metabolism of lipids can cause the development of diseases of civilization, including obesity, type 2 diabetes, and atherosclerosis [73]. In recent years, there has been a plethora of research on the regulation of circadian rhythm, the biological clock, and related dysfunctions. The circadian clock system protects lipid homeostasis; thus, any dysfunction against this background, as previously mentioned, will contribute to the development of many metabolic diseases. Individual lipid metabolic pathways, signaling associated with particular types of lipids, are intrinsically linked to the circadian rhythm. Proper architecture and sleep are crucial for maintaining proper lipid energy homeostasis.

Abnormalities occurring in the metabolic background of lipids affect the structure, composition, and permeability, with a further consequence of affecting the regulation of signaling pathways, having a bearing on the appearance of cancer cells. Despite the de novo production of fatty acids, the vast majority comes through endogenous supply, as a result of the consumption of meals rich in this energy-rich macronutrients [74]. Numerous studies show a link between impaired lipid metabolism and increased cancer development, as well as increased progression when cancer is present. Importantly, cancer cells contribute to increased lipid production of their own in order to promote the biological membrane of cancer cells, to increase membrane lipid saturation. This process is important because it affects other very important biochemical pathways. It is activated during gene expression and cell signaling; thus, there is increased proliferation, and the manifestation of this is cancer progression [75]. Lipid energy homeostasis is critically important for proper heart function. The heart is the organ in which there is the largest number of mitochondria, as a result ensuring an adequate and constant supply of energy, crucial for the proper work of such an important organ. ATP hydrolysis is a direct source of energy for mechanical activity of the cells of the heart muscle (cardiomyocytes). The amount of energy consumed by the heart in one day is about 5 kg of ATP. It is important to remember that the reserves of energy-rich compounds are scarce; therefore, there is a constant need for renewal during energy metabolism. The primary source for the cells of the myocardium are fatty acids, which cover energy requirements at the level of 60–90%, as well as lactic acids and glucose, providing energy coverage at the level of 10–40% [76]. As previously mentioned, free fatty acids (FFAs) were captured from the blood and then transported to the mitochondria. Here, the process of beta oxidation takes place, and as a result, biochemical transformations are incorporated into the tricarboxylic acid cycle in the form of acetyl-CoA. Fatty acids are more favorable in terms of obtaining energy, because from one molecule of palmitic acid, 105 molecules of ATP are formed, while from one molecule of glucose, it is only 30 molecules [77]. However, in the case of this comparison, the amount of oxygen required to produce ATP is greater when the energy source is fatty acids. This situation is not insignificant for the body, because at the moment of hypoxia, it is more beneficial for the cell to take up glucose than fatty acids, which is indeed what takes place.

Moreover, the heart is one of the organs in which NEFA uptake takes place. First of all, NEFA uptake by the heart is dependent on several steps: lipid uptake by cardiomyocytes, and the transfer from endothelial to subendothelial cells. The CD36 transporter plays an important role [78]. Interestingly, the genetic deletion of CD36 leads to reduced intracellular lipid accumulation [79].

Overexpression, on the other hand, leads to increased uptake and the oxidation of lipids [80]. Impaired cardiac energy metabolism is associated primarily with decreased fatty acid oxidation (FA) and increased glucose utilization. Studies have shown a link between deletion of the CD36 receptor, which leads to a lower utilization of free fatty acids, and increased glucose expenditure. A manifestation of such abnormalities is the faster progression of heart failure (HF). It is likely that cardiac energy metabolism and the associated fatty acid uptake by the CD36 receptor is crucial in the perspective of maintaining the homeostasis of ATP production, and thus is an important component of the treatment of HF [81]. DAG functions as an intermediary in lipid metabolism, facilitating the formation of TAG and phosphatidic acid (PA) through the enzymatic activity of diacylglycerol acyltransferases (DGATs) and diacylglycerol kinases (DGKs). This pathway is essential for sustaining lipid equilibrium and cellular energy regulation [24]. Supplementation with DAG has been investigated for its potential role in regulating body fat, lipid metabolism, and associated health issues. As a minor constituent of plant oils, DAG has been found to affect lipid metabolism in a manner distinct from TG [24]. DG supplementation has demonstrated the ability to enhance lipid metabolism by lowering serum triglyceride levels and promoting fat oxidation. Studies in diabetic patients and animal models have shown that DG intake leads to reduced serum triglycerides and improved glucose tolerance, highlighting its potential for managing hyperlipidemia and diabetes. Furthermore, DG has been linked to the increased activity of lipid metabolism enzymes, such as acyl-CoA oxidase, which may play a role in its positive effects on lipid profiles [82].

### 3.6. Clinical Significance of Different Lipid Types and Mechanisms Affecting Health

Considering the diversity of lipids, and thus the different effects on different organs, as well as the functions they perform in the human body [83], we take a closer look at by what mechanisms lipids affect the body. Historically, lipids were considered energy storage compounds and building blocks of the basic elements of a eukaryotic cell. Current data present many biochemical reactions in which lipids show indirect or direct action. Lipids are classified as molecules with hydrophobic properties and hydrophilic properties (amphiphilic molecules).

In the cell membrane, phospholipids, of which there is a later discussion, are arranged in a structure defined by a bilayer with phosphate heads, oriented towards the water, and tails, arranged in the interior [53]. This arrangement limits the contact of hydrophobic tails with water molecules, and this property provides a stable, energy-efficient arrangement. Phospholipids exhibit a multi-tropic effect on the human body; in addition to affecting the function of the cell, the use of the micelle can be seen, for example, in supplement products, in which the compound is packed in the form of micelles. The formation of micelles is energetically beneficial as it isolates the hydrophobic fatty acid tails, thereby allowing the hydrophilic phosphate head to interact with the surrounding water [84]. The properties of lipid molecules and the arrangement determine the function and morphology of lipids in living organisms [84]. Disorders related to lipid levels have been linked to diseases such as diabetes, atherosclerosis, obesity, cancer, autoimmune diseases, neurodegenerative diseases, and cardiovascular disease. Classification and division of fatty acids: SCFA—formed by the fermentation of polysaccharides, the main representatives of SCFA, and derived from the microbiome including propionic acid (C3;0), butyric acid (C4;0), and acetic acid (C2;0) ratios, whose proportions are dependent on the type of diet, diseases, and age. SCFAs are very important metabolites, affecting the modulation of the immune system, and as a result, their dysfunction is associated with inflammatory bowel diseases [85]. SCFAs are built from 1 to 6 carbon atoms in the aliphatic chain, although the main representatives of SCFAs (the previously mentioned butyric acid, acetic acid, and propionic acid) in this group also include valeric acid and capric acid [86]. Importantly, the concentration of SCFA is variable depending on the location in the proximal segment of the colon (70–140 mM), while in the distal segment (20–70 mM), this difference is due to the greater availability of substrates and water. The largest changes in concentration were observed for butyric acid [87]. An important issue is the absorption of SCFA, which settles at 95% and takes place through intestinal epithelial cells [88]. Maintaining microbiome homeostasis is critically important for properly developing the immune system. Data indicate that special attention should be paid to the first 1000 days of a child’s life. Numerous studies emphasize the role of bacterial metabolites in the lower gastrointestinal tract. The removal of bacterial flora (germ-free) in mice is associated with several immune-mediated abnormalities including a thinner epithelial mucus layer and an impaired structure of lymphoid organs [89,90]. A significant function of SCFAs in the body, confirmed in many clinical studies to date, is the trophic effect exerted on the intestinal epithelium. This action is associated with the acceleration of healing and regeneration processes. Of the three main SCFAs, butyric acid has the most beneficial effect in this regard. SCFAs play an extremely important role in maintaining the normal structure, integrity, and function of the intestine. By stimulating the growth of saprophytic flora, they inhibit the aforementioned pathogens, as well as through competition at the site of colonization [91,92]. Butyric acid and its salts appear to have the strongest anti-inflammatory effect. Sodium butyrate shows the ability to reduce IL-8 secretion, which contributes significantly to blocking the anti-inflammatory cytokine cascade at the local level [93]. This is a particularly desirable effect in chronic inflammatory conditions such as Crohn’s disease and ulcerative colitis, when butyrate is followed by a significant improvement in the intestinal mucosa. This effect, observed by clinical, endoscopic, and histological examination, involves a reduction in the number of macrophages and neutrophils in the crypts and on the surface of the intestinal epithelium and leads to the inhibition of further disease progression [91,94]. Multiple sclerosis (MS) is one of the neurodegenerative diseases. In MS, damage to the myelin sheath designed to protect nerve axons is observed. It is a progressive, debilitating disease, leading to death. One study examined the effects of propionic acid on MS, with interesting results that were very promising for patients diagnosed with multiple sclerosis. This study showed that MS patients had reduced fecal and serum PA compared to the control group. Only after 2 weeks of propionic acid supplementation were the changes observed regarding regulatory T cells increased, while there was a decrease in TH1 and TH17 cells. In addition, there was an increase in subcortical gray matter and a reduction in cerebral atrophy. Propionic acid appears to be an important component when treating patients with MS [95]. Another acid from the family of short-chain fatty acids is valeric acid (C5;0), which admittedly is not as abundant as, say, butyric acid or propionic acid, but is worth mentioning. First of all, valeric acid will affect cellular metabolism, as shown in one study. This effect is mainly related to the effect on B cells and CD 4+ effector T cells, one of the key tasks of which is the recruitment and activation of phagocytosis towards the destruction of microorganisms. Furthermore, valeric acid shows anti-inflammatory effects in suppressing the pathogenic phenotype of Th17 cells. Another important mechanism is the effect on increasing the oxidation of glucose by the previously mentioned lymphocytes, resulting in the activation of the mTOR pathway, in order to inhibit the secretion of ILF-17A and increase the expression of Il-10. Taking into account the individual functions of valeric acid, in mouse models, the reduction in diseases such as multiple sclerosis (MS), colitis, and other autoimmune diseases has been shown, in which there is a disorder dependent on Th17 cells [53]. Long-chain fatty acids are made up of 12 or more carbon atoms, between which there are two or more double bonds [96]. Fats, found in food, are the most efficient and, at the same time, concentrated source of energy, conducive to fat-soluble vitamins (A, D, E, K). Importantly, however, in addition to their energy function, long-chain fatty acids exhibit pleiotropic biological effects, especially the acids of the omega-3 family and the omega-6 family. Polyunsaturated essential fatty acids (PACs) play a key role in maintaining the body’s homeostasis. The nomenclature of the acids is related to their chemical structure. In the omega-3 family, the first double bond occurs at the third carbon from the methyl end of their backbone and is synthesized from the essential fatty acid α-linolenic acid (ALA) [97]. In contrast, acids of the omega-6 family double bond occur at the sixth carbon counting from the methyl end of the aliphatic chain and are synthesized from linoleic acid. The omega-3 acids include α linolenic acid, docosahexaenoic acid (DHA), and eicosapentaenoic acid (EPA). On the other hand, omega-6 acids include linoleic acid and arachidonic acid. It should be borne in mind that, of the four types of unsaturated acids, biological action is shown mainly by the previously mentioned omega-3 and 6. α linolenic acid (ALA) represents omega-3, while linoleic acid (LA) represents omega-6. Essential fatty acids are needed since the human body does not have the ability to synthesize EFAs, due to the lack of enzymatic systems capable of introducing double bonds at the n-3 and n-6 positions. Importantly, the body shows the ability to remodel them, with the participation of specialized enzymes. Therefore, providing an adequate supply of EFAs is key to maintaining health [53]. The antagonistic effect of omega-3 and 6 acids should be a key aspect of their selection. One of the main functions of omega-3 fatty acids is anti-inflammatory and anti-aggregation; in turn, omega-6 exhibits pro-thrombotic and pro-inflammatory effects. Ensuring an adequate supply of long-chain essential unsaturated acids is key to maintaining health and covering the body’s metabolic needs. Heart disease is the leading cause of disease worldwide, and adequate prevention seems to be an effective method to reduce deaths from cardiovascular disease. Numerous studies have demonstrated the beneficial use of n-3 PUFA, mainly by normalizing hypertriglyceridemia and preventing heart failure and atherosclerotic plaque rupture [53]. Addition, it has also been shown to reduce blood pressure, which is one of the main factors in the development of such diseases as stroke, heart attack, heart failure, and atherosclerosis. Given the strong anti-inflammatory effect, the use of omega-3 fatty acids for the prevention of atherosclerosis seems appropriate. Inflammation is an essential factor in the development of atherosclerosis, so targeting the reduction, reducing the secretion of particular pro-inflammatory cytokines, can be one of the elements of prevention, as well as the already formed changes [98]. DHA restores the function of vascular endothelial cells (disruption occurs due to damage to the blood vessel wall), reduces organ damage, and exhibits vasoprotective effects [99,100]. DHA and EPA also affect the reduction in TNF (tumor necrosis factor α) IL6 and IL-1β. Moreover, EPA and DHA show protective effects in atherosclerotic plaque rupture. PUFAs also mediate protective effects in atherosclerotic plaque rupture [101]. In one study in rats with colitis, n-3-rich fish oil administration led to the inhibition of LTB4 and PGE2 (a potent pro-inflammatory, immunosuppressive prostaglandin) production in the colon, as well as VEGFR2 and VCAM-1 in endothelial cells—further evidence of the essential contribution of EPA and DHA to cardiovascular disease [102]. Polyunsaturated essential fatty acids n-3 and n-6 are an essential component of phospholipids, which are a key component of cell membrane structure. Phospholipids ensure proper fluidity of the cell membrane and semi-permeability, which is crucial for the communication of the cell with surrounding structures. DHA n-3 acid is a key component of nervous tissue primarily of the cerebral cortex and the retina of the eye. DHA in the human brain accounts for 25–35% of all TCs; even more significantly, in the case of neurons, it is as much as 60%. Therefore, it is essential for the proper development and functioning of the nervous system. It is essential for the proper development of the baby’s brain from the third trimester of pregnancy, at a time when the fetal brain is going through a stage of intense growth. Importantly, DHA-3 is accumulated by the fetus between 25 and 40 weeks of pregnancy. The consequences of DHA-3 deficiency, especially in young children (where this need is especially crucial), cause attention-deficit hyperactivity disorder (ADHD), impaired vision (the main building block of the eye’s photoreceptors), and a reduced intelligence quotient. Deteriorated cognitive functions (problems with concentration with memory and reading comprehension) are also a common problem. DHA n-3 deficiency is also associated with skin problems, especially atopic dermatitis. Problems with cognitive function in young children are increasingly observed, of which there may be several reasons for such a phenomenon (excessive consumption of highly processed foods, weight problems, lack of exercise, deficiencies in omega-3 fatty acids, especially DHA). One study showed that the DHA supplementation of 600 mg (from microalgae oil) in children aged 7–9 years, for 16 weeks, improved skills in children who had more trouble reading [103]. DHA affects the use of cerebral cortex activity during increased attention. In one study that included boys aged 8–10, supplementation with DHA (again from microalgae oil) at a dose of 400–1200 mg per day, for a period of 8 weeks, improved activation of the dorsolateral prefrontal cortex and reduced activation in the cerebellum and occipital cortex, significantly demonstrated in both groups that were given supplementation [104]. In addition, DHA is an extremely important component of the myelin sheath, which is a kind of insulation of neurons, a key element in the case of neurodegenerative diseases in which myelin damage is observed. More than 50% of the total weight of the sheath is DHA; moreover, the fatty acids found in the brain are mainly DHA, whose value reaches 97% [105]. Importantly, the best and most bioavailable source of EPA I DHA acids is fatty marine fish in particular (salmon, herring, halibut, tuna). Despite the possible conversion of ALA to EPA I DHA, the process is not very efficient. It is estimated that the conversion of ALA-EPA is (0.2; 21%), while ALA-DHA is (0–9%) [106]. It is worth noting that a number of factors influence the degree of conversion of ALA to EPA and DHA. The action of enzymes responsible for the conversion are, in turn, desaturase—responsible for the introduction of additional unsaturated bonds—and elongase—responsible for the elongation of the carbon chain. The whole process takes place in the endoplasmic reticulum of the cells. It is worth emphasizing that the activity of the above-mentioned enzymes is dependent on ensuring an adequate supply of vitamin B6, magnesium, and zinc. Often-observed problems with glucose–insulin metabolism will also negatively affect the activity of enzymes [107]. On the other hand, the biggest problem seems to be the overconsumption of vegetable oils rich in n-6. This is due to the unfavorable ratios of n-6 to n-3 PUSFs outside (olive oil, flaxseed oil). The process of the conversion of linolenic acid n-6 and -α linoleic acid n-3 to DHA and EPA is carried out by the same enzymes. On the other hand, preference is given to acids of the omega-6 family, as a result of which the step of converting alpha linolenic acid n-3 is practically impossible, which translates into an even greater disproportion between n-6 and n-3 in the human body. It is a mistake to equate vegetable oils as the main source of n-3 PUSFs because the existing ratios of n-3 PUSFs to n-6 PUSFs are detrimental to the health of the body, especially when we consider what a significant difference there is between the expected and actual state. The quantitative ratio of n-6 to n-3 fatty acids in the typical diet of Western countries is 20–30; moreover, it is among the main risk factors for diseases such as atherosclerosis, cancer, neurodegenerative diseases, and cardiovascular diseases [107]. As a result of cyclooxygenase (COX), dienes eicosanoids are formed from arachidonic acid. They are characterized by high biological activity, even in small amounts. The main representatives are PGE2 (prostaglandin E2), which shows pro-inflammatory and immunosuppressive effects; PGI2 (prostacyclin I2), which is formed in the vascular endothelium and shows vasodilatory and anti-aggregatory effects; TXA2 (thromboxane A2), which is formed in thrombocytes and shows a strong vasoconstrictive effect; and pro-aggregation, which increases the oxygen demand of cardiac cells as a result of the influx of calcium ions into the vascular and cardiac muscle cells; a manifestation of such an action is increased contractility. Furthermore, numerous studies show their pro-cancerogenic effects through increased proliferation and proliferation, especially in breast, colorectal cancer, and prostate tumors [108]. It is worth noting that despite the increased consumption of supposedly healthy vegetable fats, the state of health in highly developed countries has not improved—quite the opposite. Just look at the statistics of the number of diseases such as neurological diseases and atherosclerosis and its complications in the form of stroke, brain, and heart attack [109].

### 3.7. Lipids in Therapeutic Diets

Lipids play a significant role in therapeutic diets, influencing various health outcomes. These diets are often designed to manage conditions like cardiovascular disease, Crohn’s disease, non-alcoholic fatty liver disease (NAFLD), and malnutrition. Therapeutic diets, like those advocated by the US National Cholesterol Education Program, aim to lower total cholesterol, triglycerides, and LDL cholesterol, playing a vital role in supporting cardiovascular health. These diets have demonstrated positive effects on lipid profiles, although their influence on glycemic indexes and blood pressure depends on the type of diet and the length of the intervention [110]. In Crohn’s disease, the composition of dietary fats plays a critical role in therapeutic outcomes. Diets rich in linoleate have been associated with higher remission rates compared to those high in oleate, highlighting the importance of dietary fat type in managing the condition effectively [111]. For NAFLD, diets high in unsaturated fatty acids, such as the Mediterranean diet, have proven effective in reducing intrahepatic lipid content and improving liver enzyme levels. These diets aid in managing liver fat accumulation and enhancing overall liver function [112,113,114]. Lipid-rich therapeutic foods can improve iron absorption, especially from poorly soluble iron compounds. This effect is advantageous in addressing malnutrition, as lipids prolong gastric residence time, potentially enhancing overall nutrient absorption [115]. Bioactive lipids, such as fatty acids and other lipid compounds, offer numerous health benefits, including the prevention of chronic diseases and the enhancement in metabolic health. Their absorption, metabolism, and health effects are largely determined by their chemical composition and structural characteristics [1]. Lipids play a significant role in drug-resistant epilepsy (DRE), with particular attention to fatty acid composition and potential therapeutic interventions. Patients with DRE show distinct alterations in blood fatty acid profiles compared to healthy individuals. They exhibit elevated levels of saturated and monounsaturated fatty acids, such as C14:0, C16:0, C18:0, and C18:1n-9, alongside reduced levels of essential n-6 and n-3 polyunsaturated fatty acids, including C20:5n-3 and C22:6n-3. These abnormalities indicate disruptions in the activity of enzymes such as elongase and desaturase, which are essential for proper fatty acid metabolism [116]. Omega-3 fatty acids, especially DHA and EPA, have demonstrated potential in decreasing seizure frequency in DRE. They are thought to modulate ion channels and lower neuronal excitability, though evidence from human clinical trials is still developing. While preclinical studies are promising, larger and more comprehensive human trials are necessary to validate the efficacy of omega-3 fatty acids as a treatment for DRE [116,117]. The relationship between lipid-lowering medications and epilepsy is complex. Genetic studies indicate that some lipid-lowering drugs may affect epilepsy risk, as higher expression of genes targeted by these medications could potentially increase the risk of developing epilepsy.

Lipids in therapeutic diets play a crucial role in managing and improving health outcomes across diverse conditions. Their impact on cardiovascular health, Crohn’s disease, NAFLD, and malnutrition emphasizes the significance of dietary fat composition in therapeutic approaches. These insights highlight the potential of customized lipid-based dietary strategies to effectively enhance health and manage diseases.

### 3.8. Debate on Saturated and Trans Fats

Recent research indicates that saturated fats may not be strongly linked to a higher risk of all-cause mortality, CVD, CHD, ischemic stroke, or type 2 diabetes. Nonetheless, some studies point to a possible association between a high consumption of saturated fats and an increased risk of death from all causes, CVD, and cancer [118]. Traditionally, reducing saturated fat consumption was advised to decrease the risk of CVD [119,120]. However, recent findings suggest that substituting saturated fats with carbohydrates may not effectively lower CVD events or mortality. Conversely, replacing saturated fats with PUFAs or MUFAs has been linked to reduced mortality and a lower risk of CVD [119,121]. Trans fats, especially those produced industrially, are connected to higher rates of all-cause mortality, CHD, and CVD mortality [118,122]. Their consumption is associated with elevated LDL cholesterol levels, a known risk factor for heart disease [121,123]. Trans fats were originally advocated as a healthier substitute for saturated fats, but this perspective shifted as research revealed their harmful health effects [124]. Present guidelines now stress the importance of minimizing trans fat consumption due to their detrimental impact on health [123]. The discussion surrounding saturated and trans fats illustrates the complexity of dietary fat recommendations. Although saturated fats may be less harmful than previously believed, substituting them with healthier options such as PUFAs and MUFAs is advantageous. In contrast, trans fats are uniformly associated with adverse health effects and should be limited in the diet. This evolving knowledge highlights the importance of nuanced dietary guidelines that take into account both the type and source of fats consumed.

### 3.9. Lipid Supplements and Functional Foods

Lipid supplements and functional foods enriched with lipids are gaining recognition for their potential health benefits, especially in the management of lipid metabolism, cardiovascular health, and metabolic disorders. Functional foods and supplements containing bioactive lipids can effectively modulate lipid metabolism and reduce adipose tissue accumulation, offering benefits for the management of obesity and type 2 diabetes [125]. Specific functional components such as omega-3 fatty acids, β-glucans, phytosterols, and vitamin E have been shown to significantly reduce lipid profiles and postprandial glucose levels, helping prevent cardiovascular diseases and metabolic disorders [126]. Functional lipids, including long-chain polyunsaturated fatty acids (LC-PUFAs), phytosterols, and omega-3 and omega-6 fatty acids, have been shown to reduce the risk of cardiovascular diseases by improving lipid profiles and lowering blood pressure [127,128,129]. Antioxidant lipid supplements, such as lycopene, astaxanthin, and beta-carotene, have shown positive effects on systolic blood pressure, LDL cholesterol, HDL cholesterol, total cholesterol, and triglycerides, contributing to a reduction in cardiovascular risk factors [130]. Functional foods and dietary supplements such as soy protein, green tea, plant sterols, probiotic yogurt, and marine-derived omega-3 fatty acids have proven effective in lowering plasma lipid levels, making them valuable tools in managing dyslipidemia [128]. Mixtures of functional foods, such as nattokinase, red yeast rice extract, and garlic, have demonstrated significant reductions in serum lipid profiles and liver enzyme levels, highlighting their potential in the treatment of hypercholesterolemia [131]. Antioxidant and probiotic/symbiotic/prebiotic supplements have shown potential in reducing liver-related markers such as ALT, AST, and LDL-C in patients with NAFLD. However, the efficacy of fatty acids and vitamin D in this context remains uncertain [132]. The development of functional foods containing bioactive lipids faces challenges related to the physical and chemical properties of these lipids, which impact product formulation, packaging, processing, and shelf-life [127]. Further research is needed to clarify the appropriate dosages, dietary intake, effectiveness, and mechanisms of action of functional lipids. Additionally, it is essential to develop reliable disease biomarkers and gain a better understanding of the long-term effects in humans [129]. Lipid supplements and functional foods containing lipids provide notable health benefits, especially in managing lipid metabolism, cardiovascular health, and metabolic disorders. Although these benefits are promising, further research is necessary to optimize their use and fully understand their long-term effects [123].

### 3.10. ESPEN Guidelines and Recommendation for Lipids

When discussing the role of fat in clinical nutrition, it is worth including information about its significance and relevance in the guidelines and recommendations of the ESPEN. ESPEN repeatedly highlights fats, their properties, and the recommended amounts based on gender, age, and the physiological condition of patients requiring specialized nutrition. According to “Lipids in the intensive care unit: Recommendations from the ESPEN Expert Group”, although enteral and PN using vegetable oils rich in linoleic acid remain common, newer lipid components like medium-chain triglycerides and olive oil have proven to be both safe and well tolerated. Nutrition enriched with fish oil (FO) is also well tolerated and offers added clinical advantages, particularly for surgical patients, thanks to its anti-inflammatory and immune-modulating properties [133]. According to ESPEN guidelines, lipids are a crucial component of PN, serving as a key energy source and providing essential fatty acids. Intravenous lipid emulsions (LEs) can be safely administered at a rate of 0.7 g/kg to 1.5 g/kg over a 12- to 24-h period [134]. Currently available lipid emulsions (LEs) for PN include various formulations: pure soybean oil (SO); a 50:50 blend of soybean oil and medium-chain triglycerides (SO/MCT); inter-esterified SO and MCTs (medium-chain triglycerides); a 20:80 combination of soybean and olive oils (SO/OO, referred to here as olive oil-based); a 40:50:10 mixture of SO, MCTs, and fish oil (SO/MCT/FO); a 30:30:25:15 blend of SO, MCTs, olive oil, and fish oil (SO/MCT/OO/FO); and pure fish oil (FO). These formulations also contain additional components, such as varying levels of phytosterols (plant-derived cholesterol-like compounds), α-tocopherol and other fat-soluble bioactives depending on the lipid source, and phospholipids—typically phosphatidylcholine (commonly known as lecithin)—used as emulsifiers [135].

According to “ESPGHAN/ESPEN/ESPR/CSPEN guidelines on pediatric parenteral nutrition: Lipids”, intravenous lipid emulsions (ILEs) are a fundamental component of PN in pediatric patients, serving as either the primary source of nutrition or as a complement to enteral feeding. In preterm infants, ILEs can be initiated immediately after birth or no later than day two of life, and if enteral feeding is discontinued, lipid administration should commence alongside PN. Parenteral lipid intake should not exceed 4 g/kg/day in preterm and term infants, while in children, the upper limit is 3 g/kg/day. To prevent essential fatty acid (EFA) deficiency, preterm infants require a lipid dosage providing at least 0.25 g/kg/day of linoleic acid (LA), which also ensures sufficient α-linolenic acid (LNA) intake. For term infants and children, a minimum LA intake of 0.1 g/kg/day is recommended, which similarly meets LNA requirements. Pure soybean oil (SO) ILEs may provide less balanced nutrition for short-term PN and are not recommended for PN lasting longer than a few days. Instead, composite ILEs with or without fish oil (FO) should be used as the first-choice treatment. Additionally, for preterm infants, ILEs should be administered using light-protected tubing to preserve lipid stability. Continuous 24 h infusions of ILEs are recommended for newborns, including preterm infants, while in home PN scenarios, ILE administration should align with the duration of other PN components. In critically ill pediatric patients, ILEs remain an essential part of PN, with composite ILEs serving as the preferred option. Although current evidence underscores the importance of timely parenteral lipid support, its effects on clinical outcomes based on timing remain unclear. In pediatric patients with sepsis, the frequent monitoring of plasma triglycerides and dose adjustments are advised in cases of hyperlipidemia. However, lipid supply should generally be maintained at levels sufficient to meet minimal EFA requirements [136].

Another important ESPEN guideline concerns the role of lipids in chronic intestinal failure in adults. In patients fully reliant on home parenteral nutrition (HPN), providing at least 1 g/kg/week of intravenous lipid emulsion (ILE) containing essential fatty acids (EFA) is recommended to prevent EFA deficiency. Transitioning from a soybean-oil-based ILE to formulations containing fish oil or olive oil can be considered safe for maintaining adequate EFA levels. No single type of lipid emulsion is universally suitable; the choice should be tailored to the individual patient’s needs. When using soybean-oil-based ILEs, the dose should not exceed 1 g/kg/day. If lipid requirements exceed this amount, alternative emulsions, such as those containing olive oil, MCT, or fish oil, are recommended. These alternatives help reduce the proportion of soybean oil, which is high in omega-6 PUFAs and phytosterols, potentially mitigating associated risks [137].

For intravenous lipids, the upper recommendation is 1 g/kg body weight/day with a tolerance of up to 1.5 g/kg/day. Excessive administration may lead to waste, storage, and even toxicity according to “ESPEN practical and partially revised guideline: Clinical nutrition in the intensive care unit” [138].

In guidelines about clinical nutrition in surgery by ESPEN, enteral nutrition (EN) and PN play equally vital roles in patients following liver transplantation. Studies have shown that using lipid emulsions containing MCT and long-chain triglycerides (LCTs) offers advantages over those with only LCTs, particularly in promoting the regeneration of the reticuloendothelial system’s function. Notably, no differences in metabolism were observed between these lipid preparations. Additionally, compared to standard treatment involving an oral diet or supplementary PN with a 20% MCT/LCT emulsion, administering an omega-3 fish oil lipid emulsion for seven days post transplantation provided significant benefits. These included reduced ischemia–reperfusion graft injury, lower infectious morbidity, and shorter hospital stays after transplantation [139].

**Figure 2:**
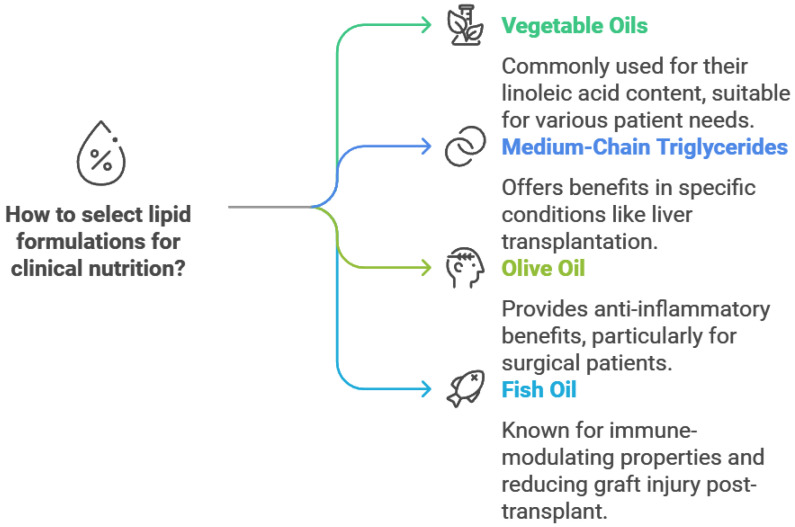
Summary of ESPEN guidelines and recommendation for lipids in clinical nutrition.

Described ESPEN guidelines are schematically summarized in Figure 2. It should be noted that these guidelines emphasize the importance of incorporating lipids into clinical nutrition strategies while accounting for individual patient conditions and nutritional requirements. The selection of lipid formulations should prioritize safety, tolerability, and clinical benefits to optimize therapeutic outcomes in diverse patient populations.

### 3.11. Implications of Lipids for Clinical Practice

An illustrative example of this is lifestyle medicine, which takes an integrated care approach to support individuals with chronic conditions such as type 2 diabetes, focusing on preventing potential complications related to diet, environmental influences, and stress management [140,141]. Managing elevated blood lipids primarily involves lifestyle modifications, particularly dietary adjustments. Strategies such as incorporating plant sterols and lowering carbohydrate consumption have been linked to decreases in LDL cholesterol and triglycerides, respectively [142]. Diets focused on therapeutic lifestyle changes, characterized by low-calorie and low-fat content, have demonstrated notable improvements in lipid profiles, including reductions in total cholesterol and LDL cholesterol [143]. Nutritional therapy that emphasizes lipid intake plays a vital role in preventing lifestyle-related diseases. Enhancing the consumption of n-3 PUFAs, such as EPA and docosahexaenoic acid (DHA), has been linked to improved insulin sensitivity and a lower risk of developing NAFLD. This underscores the critical role of lipid-focused nutrition in managing and mitigating lifestyle-related health conditions [144,145].

Although the advantages of lipids in lifestyle medicine are clear, challenges persist in standardizing lipidomics techniques and unraveling the intricate interactions of lipids within the body. Advancing the field will require the development of accurate biomarkers and further research into lipid-focused interventions to enhance patient outcomes [146].

## 4. Conclusions

The diversity of lipids affects their unique and specific biological functions. The use of selected and individual lipids is an important element in the case of fighting with various metabolic diseases. It is worth noting that many clinical studies confirm the use of lipids in various disease entities. The wider use of vegetable oils should be focused on, the negative effects of which at the cellular level is an important element in the perspective of potential reduction in order to protect health. The pleiotropic action of lipid compounds provides an opportunity for application in biomedicine; as a result, the possibility of recovery is facilitated. Increasing research on functional lipids can be expected. It is worth looking more widely at the use of vegetable oils whose negative effects at the cellular level are important in the perspective of potential reduction in order to protect health.

**Figure 3:**
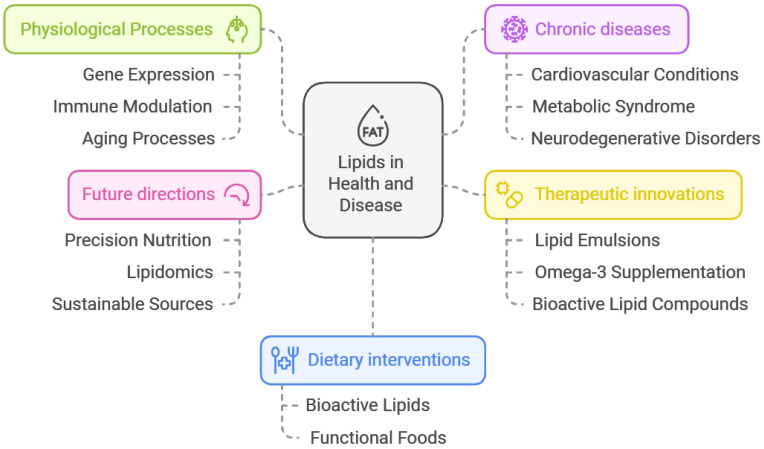
Summary of lipids in clinical nutrition and health with future directions.

This review highlights the critical importance of lipids in physiological processes and their role in the pathogenesis and treatment of chronic diseases such as cardiovascular conditions, metabolic syndrome, and neurodegenerative disorders, which is summarized in Figure 3.

Discoveries regarding their regulatory effects on gene expression, immune modulation, and aging processes open new therapeutic opportunities. Innovations in the clinical use of lipids such as lipid emulsions, omega-3 supplementation, and bioactive lipid compounds demonstrate their potential to improve outcomes in conditions like Crohn’s disease, non-alcoholic fatty liver disease, and drug-resistant epilepsy. Properly designed nutritional interventions, especially those aligned with ESPEN guidelines, offer promising perspectives for personalized medicine.

The balance between different types of lipids, including saturated, unsaturated, and trans fats, remains pivotal. Unsaturated fatty acids, particularly omega-3 and omega-6, support cardiovascular and neurological health, whereas the excessive intake of saturated and trans fats increases disease risk. Encouraging dietary changes that emphasize bioactive lipids and functional foods can bring substantial health benefits. However, debates about the effects of saturated fats and the technical challenges of incorporating lipids into functional products highlight the need for further research and innovation in this area.

The future of lipid science lies in the development of precision nutrition and lipidomics, enabling the creation of targeted dietary interventions tailored to individual metabolic needs and health objectives. Continued research into the mechanisms of bioactive lipids and their synergistic effects in functional foods could unlock novel therapeutic potential. Refining and harmonizing clinical guidelines based on the latest scientific evidence will ensure optimal patient care. Additionally, developing sustainable sources of high-quality lipids, particularly marine-derived omega-3s, and ensuring their global accessibility are essential for advancing public health.

## Data Availability

The original contributions presented in the study are included in the article, further inquiries can be directed to the corresponding author.

## Supplementary Materials

The following supporting information can be downloaded at https://www.mdpi.com/article/10.3390/foods14030473/s1: Table S1: (SANRA analysis for “Lipids in clinical nutrition and health: narrative review and dietary recommendations”).
